# Comparing the prognostic value of geriatric health indicators: a population-based study

**DOI:** 10.1186/s12916-019-1418-2

**Published:** 2019-10-02

**Authors:** Alberto Zucchelli, Davide L. Vetrano, Giulia Grande, Amaia Calderón-Larrañaga, Laura Fratiglioni, Alessandra Marengoni, Debora Rizzuto

**Affiliations:** 1 Aging Research Center, Department of Neurobiology, Care Sciences and Society, Karolinska Institutet and Stockholm University, 11330 Stockholm, Sweden; 2 Department of Clinical and Experimental Sciences, University of Brescia, Viale Europa 11, 25121 Brescia, Italy; 3 Centro di Medicina dell’Invecchiamento, IRCCS Fondazione Policlinico “A. Gemelli” and Catholic University of Rome, 00168 Rome, Italy; 4 Stockholm Gerontology Research Center, 11330 Stockholm, Sweden

**Keywords:** Health indicators, Older persons, Prognosis

## Abstract

**Background:**

The identification of individuals at increased risk of poor health-related outcomes is a priority. Geriatric research has proposed several indicators shown to be associated with these outcomes, but a head-to-head comparison of their predictive accuracy is still lacking. We therefore aimed to compare the accuracy of five geriatric health indicators in predicting different outcomes among older persons: frailty index (FI), frailty phenotype (FP), walking speed (WS), multimorbidity, and a summary score including clinical diagnoses, functioning, and disability (the Health Assessment Tool; HAT).

**Methods:**

Data were retrieved from the Swedish National Study on Aging and Care in Kungsholmen, an ongoing longitudinal study including 3363 people aged 60+. To inspect the accuracy of geriatric health indicators, we employed areas under the receiver operating characteristic curve (AUC) for the prediction of 3-year and 5-year mortality, 1-year and 3-year unplanned hospitalizations (1+), and contacts with healthcare providers in the 6 months before and after baseline evaluation (2+).

**Results:**

FI, WS, and HAT showed the best accuracy in the prediction of mortality [AUC(95%CI) for 3-year mortality 0.84 (0.82–0.86), 0.85 (0.83–0.87), 0.87 (0.85–0.88) and AUC(95%CI) for 5-year mortality 0.84 (0.82–0.86), 0.85 (0.83–0.86), 0.86 (0.85–0.88), respectively]. Unplanned hospitalizations were better predicted by the FI [AUC(95%CI) 1-year 0.73 (0.71–0.76); 3-year 0.72 (0.70–0.73)] and HAT [AUC(95%CI) 1-year 0.73 (0.71–0.75); 3-year 0.71 (0.69–0.73)]. The most accurate predictor of multiple contacts with healthcare providers was multimorbidity [AUC(95%CI) 0.67 (0.65–0.68)]. Predictions were generally less accurate among younger individuals (< 78 years old).

**Conclusion:**

Specific geriatric health indicators predict clinical outcomes with different accuracy. Comprehensive indicators (HAT, FI, WS) perform better in predicting mortality and hospitalization. Multimorbidity exhibits the best accuracy in the prediction of multiple contacts with providers.

**Electronic supplementary material:**

The online version of this article (10.1186/s12916-019-1418-2) contains supplementary material, which is available to authorized users.

## Background

The identification of individuals at increased risk of poor health-related outcomes is a clinical and public health priority. Indeed, risk stratification plays a pivotal role in medical decision-making, public resource allocation, and research [1, 2]. For example, unplanned hospitalizations, which are a major driver of healthcare costs, often lead to disability onset or progression [3, 4] and delirium [5, 6], preventing older adults from being discharged home. The identification of older persons at increased risk of unplanned hospital admissions could help to better target preventive strategies [7] (i.e. therapeutic review) toward specific groups of patients.

Accomplishing such a task is particularly critical among older persons. In fact, persons older than 60 are among the most strenuous users of healthcare resources [8, 9], and their number is expected to double worldwide by 2050 [10]. Indeed, a noteworthy variability is found among older persons, even of the same age, in terms of functional and cognitive performance, number and severity of chronic diseases, quality of life, and prognosis [11, 12].

In the last decades, researchers in geriatrics have proposed several indicators shown to be strongly associated with the development of poor health-associated outcomes, such as death and unplanned hospitalizations. The co-occurrence of multiple chronic conditions in the same individual (multimorbidity), for example, has a strong impact on health, higher than that expected by simply summing diseases [13]. Frailty, a state of increased vulnerability to stressors due to poor resolution of homeostasis [14], is another concept that gained recent recognition because of its prognostic value, even beyond the borders of geriatric practice [15, 16]. Furthermore, simple functional measures, such as the evaluation of normal pace walking speed, have been shown to be strongly associated with survival [17]. Lastly, summary scores evaluating multiple domains have been shown to have high predictive accuracy [18, 19].

These indicators differ not only in their theoretical foundation, but also in their operationalization. For example, while a general consensus on the definition of frailty has been reached [20], several ways to assess it in clinical practice and research are in use [14]. Furthermore, while these indicators have been validated in various cohorts [21–23], a head-to-head comparison of their accuracy in the prediction of different outcomes is still lacking. Such studies are of particular interest, as they may allow clinicians (as well as researchers and policy makers) to choose the most suitable predictive tool according to aims, needs, and data availability.

Thus, the aim of this study is to compare the accuracy of five geriatric health indicators (the frailty index, the frailty phenotype, multimorbidity, walking speed, and a summary score—the Health Assessment Tool) in the prediction of mortality, unplanned hospitalizations, and multiple contacts with healthcare providers.

## Methods

### Study population

Data were gathered from the Swedish National Study on Aging and Care in Kungsholmen (SNAC-K). SNAC-K is an ongoing population-based study, started in 2001. Individuals aged 60+ living in the central area of Stockholm (Sweden), either at home or in institutions, were asked to participate in the study. A comprehensive assessment using standard questionnaires, medical examinations, and interviews was performed to retrieve demographic, clinical, and functional measures of the 3363 (response rate 73.3%) persons enrolled. Data from neuropsychological assessments and physical tests were also collected, as elsewhere described [24]. Every wave of the study was approved by the Regional Ethical Review Board in Stockholm, Sweden. Written informed consent was obtained from each participant, or from a proxy, in case of cognitive impairment. The public or patients were not involved during the development of this study: anyhow, we plan to disseminate the findings of this research to participants of SNAC-K and to the public.

### Geriatric health indicators

#### Frailty index (FI)

The frailty index is a commonly employed measure of frailty, firstly proposed by Rockwood et al. [25]. It is based on the ratio (range 0–1) between the number of deficits (i.e. signs, symptoms, diseases, biomarkers, functional status, physical performance indicators) exhibited by the individual and the total number of potential deficits taken into consideration by researchers. In SNAC-K, two geriatricians (DLV and AZ) selected 45 variables (Additional file 1: Table S1) and re-codified them, in accordance with the recommendations provided by Searle et al. [26]. For baseline description purposes, participants were considered frail if they exhibit a FI ≥ 0.25, robust with a FI ≤ 0.08, and pre-frail in between, as previously reported [27]. The frailty index was considered missing if two or more variables were not available (*N* = 348).

#### Frailty phenotype (FP)

The frailty phenotype is a commonly used and validated operational definition of physical frailty, originally proposed by Fried et al. [28]. It evaluates five criteria: slow walking speed, low grip strength, unintentional weight loss, exhaustion, and low physical activity (the operationalization carried out in SNAC-K is available elsewhere [29]). For baseline description purposes, individuals meeting at least three criteria were considered frail, and those meeting one or two criteria were considered pre-frail, while the remaining were considered robust. Values were missing for 599 people in at least one criterion.

#### Multimorbidity

In SNAC-K, diseases were coded in accordance with the International Classification of Diseases 10th edition. Diagnoses were ascertained by physicians based on medical history, medical records, physical examinations, and instrumental and laboratory analyses. For baseline description purposes, we defined multimorbidity as the count of chronic conditions, based on 60 disease categories identified by Calderon-Larranaga et al. [30]. To examine the distribution of multimorbidity in our population, we used the cut-off of two or more chronic diseases.

#### Walking speed (WS)

In SNAC-K, a nurse noted the time needed for the participant to complete a 6-m straight path, walking at usual pace. Participants were allowed to use walking aids but had to complete the path without help. In case of inability to complete the path, a walking speed of zero was recorded. For those who self-reported slow walking speed or in case of at-home assessment, a 2.4-m path was used. For baseline description purposes, a WS cut-off of < 0.8 m/s was used to identify slow walking speed in our study population, as previously suggested [17].

#### Health Assessment Tool (HAT)

Proposed by our group [18], HAT is a summary score evaluating five characteristics: walking speed, Mini-Mental State Examination (MMSE) score, limitations in instrumental activities of daily living, limitations in basic activities of daily living, and count of chronic diseases. HAT was built regressing these characteristics against the latent variable “health status” using a nominal response model (more details are available in the appendix of the original article [18]), obtaining a score ranging from 0 (poor health) to 10 (good health). It has been shown to be reliable over time and to adequately predict different adverse outcomes [18, 31]. For baseline description purposes, poor health status was considered for individuals with a HAT score ≤ 3.3, while good health was considered for those with a HAT score ≥ 6.6. Data were missing for eight people.

### Outcomes

Vital status was retrieved within 3 and 5 years of follow-up using the Swedish Cause of Death Register [32]. The Stockholm County Council Register (as part of the National Patient Register [33, 34]) was used to gather data on hospitalizations and contacts with outpatient care providers (i.e. visits to both primary and specialist care), as previously described [18]. These registers contain information on the type of admission (i.e. planned or unplanned), among others. We defined “acute hospitalization” as experiencing at least one unplanned admission during the first year or the first 3 years after the baseline assessment. “Multiple provider contacts” was defined as having multiple outpatient visits in the 6 months prior and after the baseline assessment. We used the median number of planned outpatient visits (i.e. 2) as the cut-off.

### Other measures

Education level was measured as the highest degree obtained. Cognitive status was assessed using the MMSE score (both as a continuous variable and using a cut-off of 24 [35]). Disability was defined as being impaired in at least one out of six basic activities of daily living [36].

### Statistical analyses

To assess the accuracy of the different geriatric health indicators, we used the area under the receiver operating characteristic curve (AUC). In this paper, we employed the AUC as measure of predictive accuracy, since it allows to simultaneously consider the sensitivity and specificity of a continuous variable in the prediction of an outcome*.* The AUC was obtained using non-parametric ROC analysis [37], including the different indicators as continuous variables. The analyses were repeated stratifying by age, using a cut-off of 78 years, the median age of our study population. To compare the average scores of the different indicators across individuals of the same age, the raw scores were standardized into *z*-scores, using the baseline mean and standard deviation of the population. The analyses were conducted on 10 imputed datasets performing multiple imputation by chained equations. For those people for whom data on the health indicators were missing (28.4%), we created an indicator variable. This variable was equal to 1 if a given observation was missing in any health indicators and to 0 otherwise. We performed logistic regression with missing value as the outcome to test whether any of the other variables were associated with the probability to be missing (Additional file 2: Table S2). These variables were used in the imputation process. For the main analyses, pooled estimates were calculated according to Rubin’s rule [38]. The same analyses were conducted in the complete case sample (71.6%), showing consistent results in terms of direction and magnitude (Additional file 3: Table S3). All analyses were performed using Stata 15 (Stata Corp, Texas, USA), with an alpha level of .05.

## Results

The baseline characteristics of the study population are shown in Table 1: the mean age was 74.7 (standard deviation, SD 11.2) and 2182 (65%) participants were female. Older (i.e. ≥ 78 years, *N* = 1581) individuals were more likely to be female, less educated, and affected by disability, while younger participants were more likely to have better cognitive performance (all *p* < 0.001).

**Table 1:** Baseline characteristics of the study population, stratified by age

	< 78 years old *N* = 1782 (53.0%)	≥ 78 years old *N* = 1581 (47.0%)	*p* value	Total *N* = 3363
Age, mean (SD)	65.5 (4.8)	85.1 (6.1)	< 0.001	74.7 (11.2)
Sex (female), *n* (%)	1024 (57.4)	1158 (73.2)	< 0.001	2182 (64.9)
Education*				
Elementary, *n* (%)	154 (8.6)	451 (28.5)	< 0.001	590 (17.7)
High school, *n* (%)	817 (45.9)	848 (53.7)		1651 (49.6)
University, *n* (%)	811 (45.5)	282 (17.8)		1090 (32.7)
MMSE, mean (SD)	29.0 (2.1)	25.2 (7.4)	< 0.001	27.6 (4.9)
MMSE < 24, *N* (%)	25 (1.4)	348 (22.0)	< 0.001	462 (14.7)
≥ 1 impaired ADL, *n* (%)	26 (1.5)	303 (19.2)	< 0.001	283 (8.6)
Frailty index				
≤ 0.08, *n* (%)	1390 (78.0)	436 (27.6)	< 0.001	1826 (54.3)
0.08–0.25, *n* (%)	375 (21.0)	856 (54.1)		1231 (36.6)
≥ 0.25, *n* (%)	17 (1.0)	289 (18.3)		306 (9.1)
Median (IQR)	0.04 (0.05)	0.12 (0.14)	< 0.001	0.07 (0.09)
Frailty phenotype				
Robust, *n* (%)	972 (54.6)	319 (20.2)	< 0.001	1291 (38.4)
Pre-frail, *n* (%)	738 (41.4)	681 (43.0)		1419 (42.2)
Frail, *n* (%)	72 (4.0)	581 (36.8)		653 (19.4)
Health assessment tool				
≥ 6.6, *n* (%)	1672 (93.8)	792 (50.1)		2464 (73.3)
3.3–6.6, *n* (%)	94 (5.3)	506 (32.0)	< 0.001	600 (17.8)
≤ 3.3, *n* (%)	16 (0.9)	283 (17.9)		299 (8.9)
Median (IQR)	9 (0.8)	6.5 (4.4)	< 0.001	8.6 (2.6)
Multimorbidity				
0 chronic dis., *n* (%)	97 (5.4)	9 (0.6)	< 0.001	106 (3.1)
1 chronic dis., *n* (%)	289 (16.2)	37 (2.3)		326 (9.7)
2+ chronic dis., *n* (%)	1396 (78.4)	1535 (97.1)		2931 (87.1)
Median (IQR)	3 (2)	5 (4)	< 0.001	4 (3)
Walking speed				
< 1.0 m/s, *n* (%)	267 (15.0)	1112 (70.3)	< 0.001	1379 (50.0)
< 0.8 m/s, *n* (%)	141 (7.9)	890 (56.3)	< 0.001	1031 (30.7)
Median (IQR)	1.2 (0.5)	0.6 (0.6)	< 0.001	1 (0.6)

*Abbreviations*: *n* number, *SD* standard deviation, *IQR* interquartile range, *MMSE* Mini-Mental State Examination

**Fig. 1:**
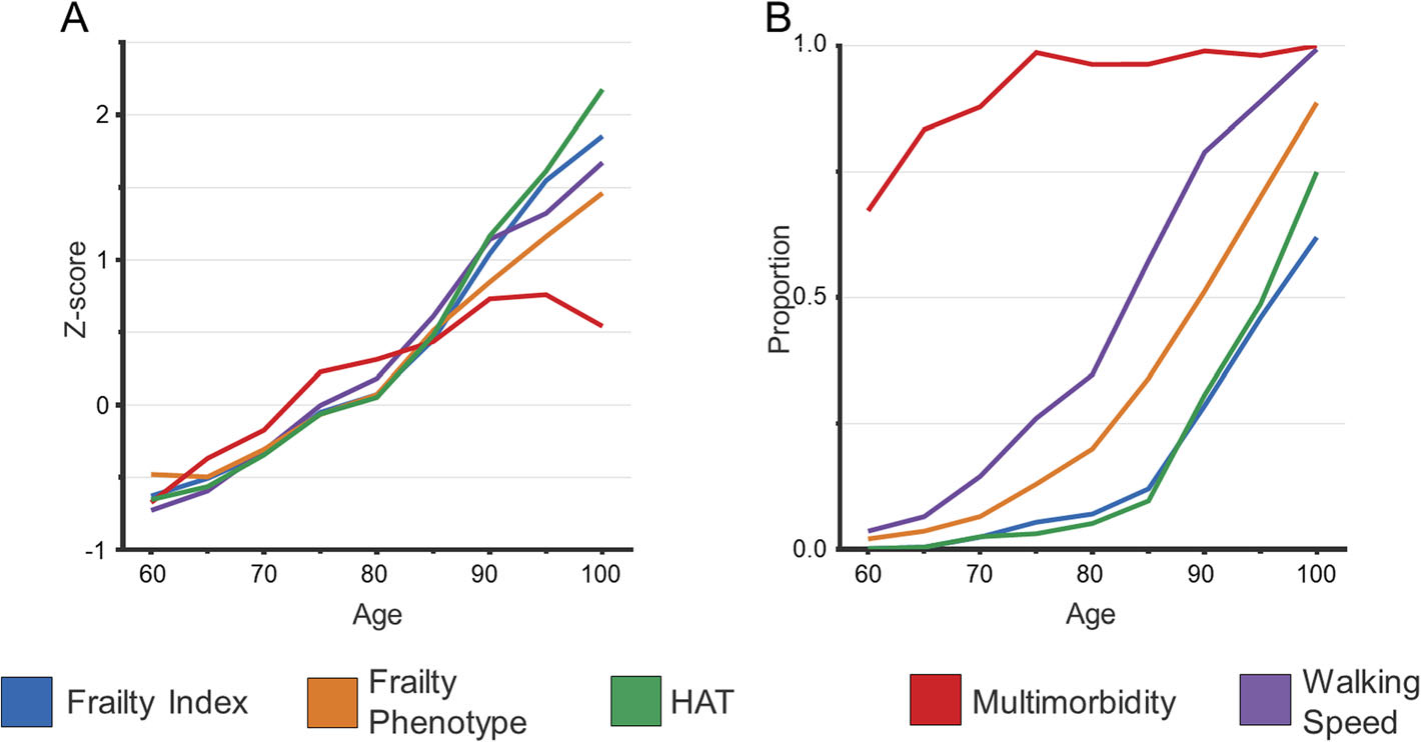
**a** Comparison of standardized indicator scores across age groups at baseline (HAT and WS were inverted to allow comparison). **b** proportion of individuals characterized by frailty index ≥ 0.25, frail phenotype, HAT ≤ 3.3, multimorbidity (2+ chronic diseases), and WS < 0.8 m/s in different age groups at baseline

The scores for all indicators were worse among older individuals, as shown in Table 1 and Fig. 1a, with the exception of the count of chronic conditions, which exhibited a plateau and a subsequent slight decline after the age of 90 years. The proportion of individuals characterized by poor health according to HAT (≤ 3.3) and of those frail according to the FI (≥ 0.25) was similar across all ages (Fig. 1b). The proportion of persons with slow WS (< 0.8 m/s) and of those considered frail according to the FP steeply increased after the age of 80 years.

**Fig. 2:**
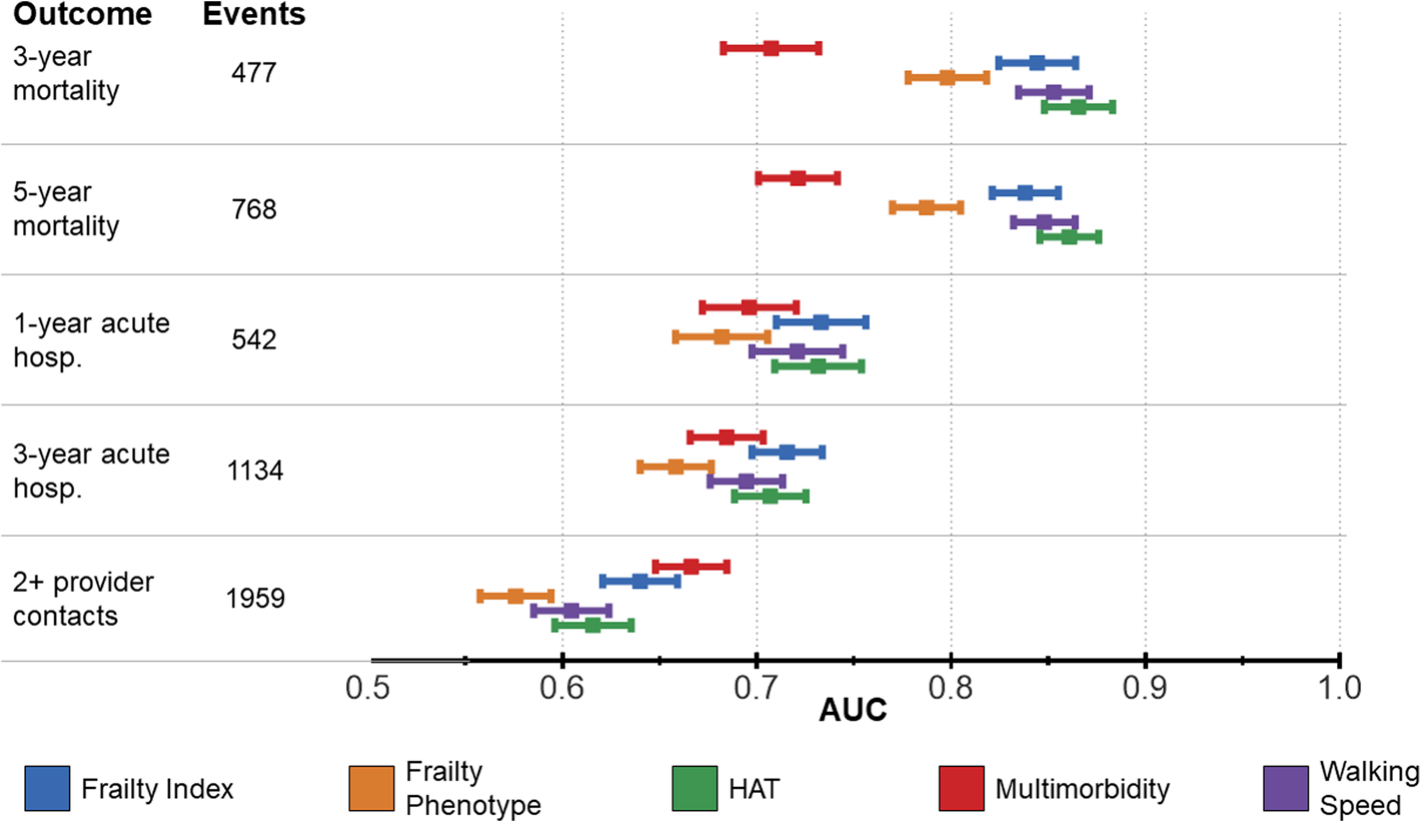
Comparison between areas under the ROC curve (AUCs) of different indicators in the SNAC-K population (*n* = 3363). HAT: Health Assessment Tool

The mean follow-up time in our study was 4.41 years. Figure 2 (and Additional file 4: Table S4 and Additional file 5: Figure S1) depicts the predictive accuracy (AUC: area under the ROC curve) of the different indicators.

### Mortality

In our study population, 477 participants (14.2%) died in the first 3 years of follow-up and another 291 in the subsequent 2 years (5-year mortality 22.8%). All indicators, with the exclusion of MM, predicted mortality with AUCs higher than 0.75: FP was the least performing indicator [3-year mortality AUC (95%CI) 0.80 (0.78–0.82); 5-year mortality AUC (95%CI) 0.79 (0.77–0.80)], while HAT showed the best AUCs [3-year mortality AUC (95%CI) 0.87 (0.85–0.88); 5-year mortality AUC (95%CI) 0.86 (0.85–0.88)]. Mortality was predicted with similar AUCs by the FI [3-year mortality AUC (95%CI) 0.84 (0.82–0.86); 5-year mortality AUC (95%CI) 0.84 (0.82–0.86)] and WS [3-year mortality AUC (95%CI) 0.85 (0.83–0.87); 5-year mortality AUC (95%CI) 0.85 (0.83–0.86)]. MM showed the worst AUC overall [3-year mortality AUC (95%CI) 0.71 (0.68–0.73)].

### Acute hospitalization

The 16.1% (*N* = 542) of our sample experienced at least one unplanned hospitalization in the first year of follow-up, while 1134 participants (33.7%) had one or more unplanned hospitalizations in the first 3 years following baseline assessment. Indicators exhibited AUCs ranging from 0.66 (0.64–0.68) [AUC(95%CI) for FP in the prediction of 3-year unplanned hospitalization] to 0.73 (0.71–0.76) [AUC(95%CI) for FI in the prediction of 1-year unplanned hospitalization].

### Multiple provider contacts

The number of individuals who had at least two contacts with care providers in the 6 months prior and after the baseline assessment was 1959 (58.2%). Among the outcomes considered, “multiple provider contacts” was predicted with the lowest AUCs. The best AUC (95% CI) was exhibited by MM 0.67 (0.65–0.68).

### Age-stratified analyses

**Fig. 3:**
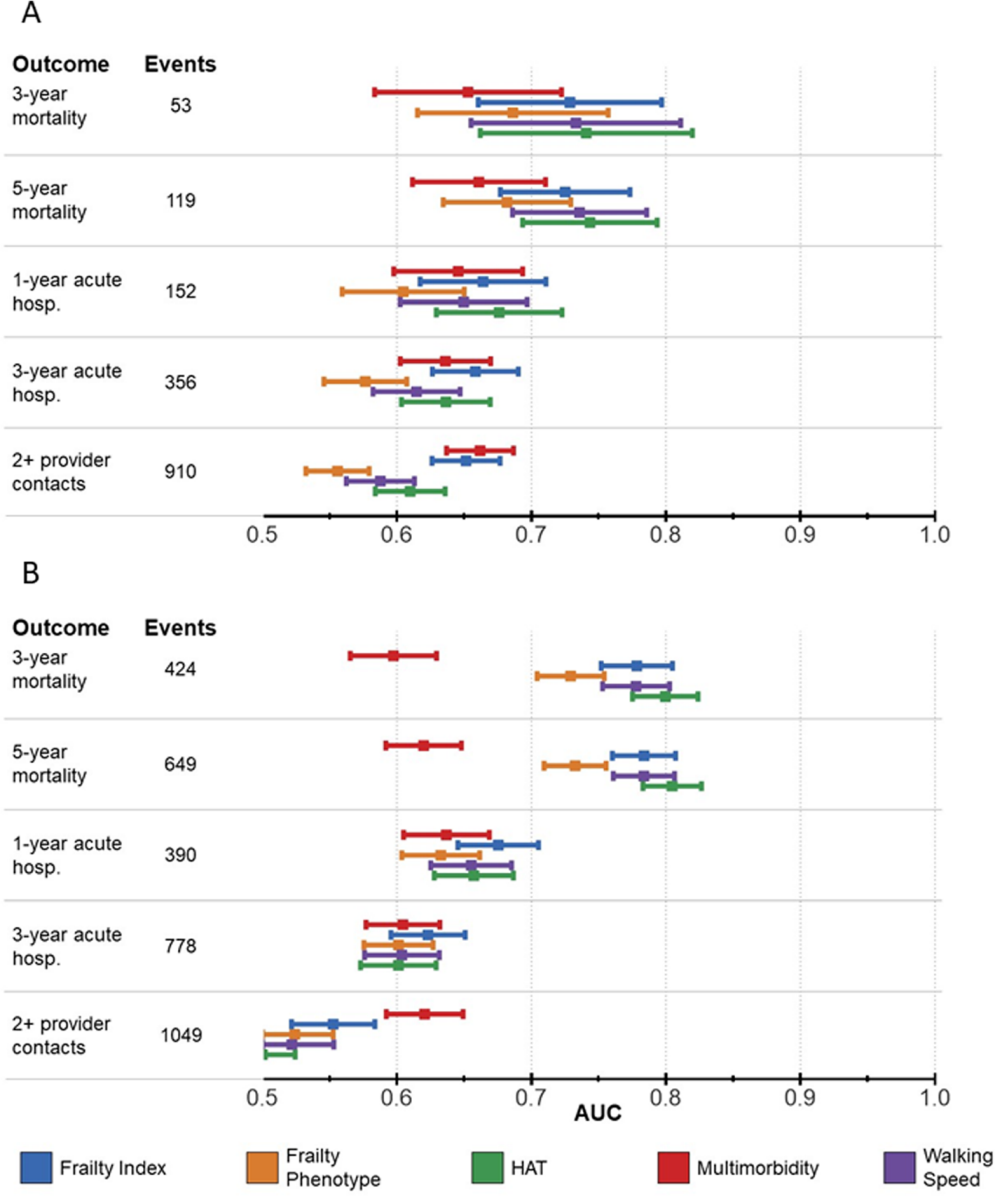
Comparison between areas under the ROC curve (AUCs) of different indicators in **a** young older adults (< 78 years old) and **b** oldest old (≥ 78 years old). HAT: Health Assessment Tool

AUCs for mortality were lower among younger individuals than among older ones, as shown in Fig. 3 (and Additional file 6: Table S5), although most of the confidence intervals were overlapping. Among younger individuals, HAT, FI, and WS showed a trend of increased accuracy in predicting mortality and unplanned hospitalization. Multimorbidity and FI predicted provider contacts with similar accuracy among younger and older individuals.

Sensitivity analyses conducted on the complete case dataset showed similar results in terms of magnitude and direction. Most indicators exhibited similar AUCs for the prediction of all outcomes, with the exception of FP and FI that showed a slightly lower predictive performance in the complete case analysis, compared to the main analysis (Additional file 3: Table S3).

## Discussion

All geriatric health indicators showed an AUC ≥ 0.70 in the prediction of mortality, while they were less accurate in predicting unplanned hospitalization and contact with multiple providers. Besides, important differences were observed in the prediction of one same clinical outcome by the different indicators. AUCs were lower among younger old persons for all indicators, with the exception of multimorbidity. HAT, WS, and FI were the most accurate predictors of mortality and unplanned hospitalization, while multimorbidity showed the highest AUCs in the prediction of contact with multiple healthcare providers.

Our findings are in line with the literature that reports AUCs ≥ 0.80 for the prediction of mortality using the FI [27, 39, 40]. Previous studies showed a prognostic accuracy for the FP ranging between 0.70 [40, 41] and 0.75 [42], although a significant variability in the assessment of the five phenotypical criteria is present. Ritt et al. [42] reported an AUC of 0.50 in the prediction of unplanned hospitalizations using the FP: the fact that the assessment was conducted in routine clinical practice conditions and the short follow-up (i.e. 6 months) might explain the difference with our findings. Several different multi-domain scores have been proposed in the previous years: despite the noteworthy variability in the variables included, reported AUCs for the prediction of unplanned hospitalization were generally higher than 0.70 [43].

Our results confirm the ability of physical function to accurately predict poor health outcomes among older individuals [17, 44–47]. Several studies suggest that disability and functional measures are strongly associated with poor health-related outcomes among older adults [17, 48, 49]. The combination of physical function and other domains, such as cognition [50, 51] or the severity of a pre-defined number of chronic conditions [19], has already been shown to help better stratify older individuals with poor prognosis. In our study, comprehensive indicators (FI and HAT) exhibited a minor but significantly higher AUCs for mortality and hospitalization, when compared to a single functional measure (WS). Different studies compared the accuracy in the prediction of mortality of physical functional indicators, such as the FP, and more comprehensive ones, such as the FI, showing different results. Our findings confirm the results of Ritt et al. [39] and Wigadgo et al. [52], who found that FP exhibited a lower discriminative performance than FI in hospitalized and community-dwelling adults. Anyhow, Li et al. [53] found similar AUCs for these two indicators. The differences with our results might be explained by the fact that in this last study, all phenotypical criteria were derived from the questions of the Short Form Survey (SF-36) and not by directly assessing walking speed or grip strength. Probably, comprehensive indicators benefit from the diversity of the information taken into account, with the inclusion of measures corresponding to different domains [51].

Interestingly, our results showed that WS alone exhibited higher AUCs for every outcome when compared with FP, despite the inclusion of walking speed among its criteria. Walking speed has been shown to be a reliable proxy of physical frailty [54, 55]: this might suggest that gait speed already provides a consistent part of the details captured by this operationalization of frailty. On the other hand, walking speed cut-offs employed for frailty phenotypical criteria (lowest quintile, adjusted by sex and height [28]) are particularly strict. While this seems to improve the specificity of FP, it might negatively affect its sensitivity [52] and, thereby, its AUC.

Furthermore, our study confirms that the simple count of chronic diseases is the most accurate indicator in predicting the use of healthcare resources, but is not as reliable in the prediction of mortality, as already described by previous studies [56]. Indeed, diagnoses—more than frailty and mobility impairment—seem to trigger clinical consultations. Previous studies already showed that increased mortality risk among persons affected by multimorbidity is probably due to a limited number of index diseases, rather than to the accumulation of chronic conditions [57]. Specific clusters of multimorbidity and the speed of accumulation—rather than the simple number—of chronic diseases have been shown to be reliably associated with several negative outcomes [58–61].

Having multiple contacts with care providers was poorly predicted by the studied indicators compared to other outcomes. Several factors might influence the number of contacts with providers, beyond people’s healthcare needs: behavioural and psychological traits, distance from the provider’s office, as well as social support, economical, and economical and insurance statuses, among others [62–65]. The studied indicators do not evaluate these aspects. Our findings highlight the need for more accurate tools to predict outpatient healthcare use.

Finally, our findings show a general trend of lower predictive accuracy for mortality when the indicators were applied to younger persons. It is likely that a higher functional resilience among younger individuals might explain the inability of currently used indicators to accurately predict poor outcomes among this subset of individuals. These results strengthen the need for a reliable tool, able to capture vulnerability to poor outcomes even among younger old individuals.

The results of the present study should be read in light of some limitations. All indicators were assessed at baseline: change of status during the follow-up might have affected the estimation of the predictive accuracy. Furthermore, minor differences with the original operationalization of some indicators exist and are related to data availability in SNAC-K. In addition, as previously described [11], the SNAC-K population is highly educated and wealthy: this might limit the generalizability of our findings. Anyhow, this issue might play a minor role because our main aim was to investigate the accuracy of different health indicators, which are based on participants’ clinical and functional characteristics. Furthermore, we found the prevalence of MM, WS, and FP to be similar to those described in previous studies [55, 66, 67]. Our study has also several major strengths. Firstly, we developed all indicators using variables derived from an in-depth and comprehensive assessment, conducted by physicians and nurses [24]. Furthermore, outcomes were retrieved from national registers, minimizing the risk of loss of information. Lastly, all indicators were built using the same data, allowing therefore a direct comparison of their predictive accuracy. Indeed, to the best of our knowledge, this is the first study directly comparing the accuracy of several indicators commonly used in geriatric research and practice for the prediction of different clinical outcomes.

### Implications

Physicians might employ indicators exhibiting a high prognostic value to better tailor diagnostic and therapeutic decisions. For example, older persons with low life expectancy benefit from therapeutic revisions aimed to control symptoms and improve quality of life [68, 69] and from the avoidance of screening tests that might lead to overdiagnosis [70]. Furthermore, high accuracy indicators might also help to prompt discussion between physicians and patients about preferences in late life [71]. The identification of older persons at increased risk of unplanned hospitalizations might be used in the clinic to plan interventions proven to lower such risk, such as more strict follow-ups [72, 73].

Healthcare policy makers could employ information regarding patients’ risk of poor health-related outcomes (such as death and hospitalizations) to better allocate resources. For example, accurately identifying individuals with decreased life expectancy is important for the integration of palliative care in modern healthcare systems [74]. Moreover, several interventions have been shown to decrease the number of hospitalizations [75, 76]: better defining the share of the population at risk of such events might enhance the effectiveness of these strategies. Furthermore, our findings showed that the count of chronic diseases could be used to predict an increased number of outpatient visits.

The indicators considered in our study might be employed according to data availability. For example, WS has already been proposed as a simple measure to be evaluated in clinical practice [77, 78], while the FI might be easily calculated from electronic clinical records [79]. HAT is based on measures easily available in clinical settings [30].

## Conclusions

Despite their different theoretical background and practical construction, HAT, WS, and FI were the most accurate predictors of mortality and unplanned hospitalizations in a population of older adults. On the other hand, multimorbidity was the most accurate predictor of contact with multiple providers. The accuracy of the considered indicators was generally lower among younger old persons compared to older ones. Different assessment tools can be used in different circumstances to support physicians during their decision-making process. Some of these tools may also be used to forecast future use of healthcare resources, including both hospital-based and outpatient services.

## Additional files

Additional file 1: **Table S1.** Deficits included in the frailty index. (DOCX 13 kb)

Additional file 2: **Table S2.** Variables associated with indicator variable of missing values used during multiple imputation process. (DOCX 13 kb)

Additional file 3: **Table S3.** Areas under ROC curves for different indicators – complete dataset analyses. (DOCX 13 kb)

Additional file 4: **Table S4.** Areas under ROC curves for different indicators – imputed dataset analyses. (DOCX 13 kb)

Additional file 5: **Figure S1.** ROC curves comparison. (JPG 241 kb)

Additional file 6: **Table S5.** Areas under ROC curves for different indicators – analyses stratified by age. (DOCX 15 kb)

## Data Availability

Data are from the SNAC-K project, a population-based study on ageing and dementia (http://www.snac-k.se/). Access to these original data is available to the research community upon approval by the SNAC-K data management and maintenance committee. Applications for accessing these data can be submitted to Maria Wahlberg (Maria.Wahlberg@ki.se) at the Aging Research Center, Karolinska Institutet.

## Abbreviations

AUC: Area under the receiver operating characteristic curve
FI: Frailty index
FP: Frailty phenotype
HAT: Health Assessment Tool
SD: Standard deviation
WS: Walking speed
