# Supplementary material for: Comparing the prognostic value of geriatric health indicators: a population-based study
Source: BMC Med. 2019 Oct 2;17:185. doi: 10.1186/s12916-019-1418-2 (PMC6774220; doi:10.1186/s12916-019-1418-2)
Supplement: Supplementary file 1 — Table S1. Deficits included in the frailty index. (DOCX 13 kb) [file 12916_2019_1418_MOESM1_ESM.docx]

**Table S1**: operationalization of the frailty index in SNAC-K.

| Hypertension | 1 if present, 0 if absent |
| --- | --- |
| Thyroid disorders | 1 if present, 0 if absent |
| Anaemia | 1 if present, 0 if absent |
| Atrial Fibrillation | 1 if present, 0 if absent |
| Blindness and other visual impairments | 1 if present, 0 if absent |
| Cerebrovascular diseases | 1 if present, 0 if absent |
| Chronic kidney disease | 1 if present, 0 if absent |
| Chronic Obstructive Pulmonary Disease, emphysema and chronic bronchitis | 1 if present, 0 if absent |
| Deafness and other hearing impairments | 1 if present, 0 if absent |
| Dementia | 1 if present, 0 if absent |
| Depression and other mood disorders | 1 if present, 0 if absent |
| Diabetes | 1 if present, 0 if absent |
| Heart failure | 1 if present, 0 if absent |
| Inflammatory bowel diseases | 1 if present, 0 if absent |
| Ischemic heart disease | 1 if present, 0 if absent |
| Osteoporosis | 1 if present, 0 if absent |
| Parkinson disease and parkinsonisms | 1 if present, 0 if absent |
| Peripheral arterial diseases | 1 if present, 0 if absent |
| Peripheral neuropathies | 1 if present, 0 if absent |
| Sleep disorders | 1 if present, 0 if absent |
| Solid neoplasm | 1 if present, 0 if absent |
| Inability to autonomously take and prepare medications | 1 if present, 0 if absent |
| Inability to autonomously shop | 1 if present, 0 if absent |
| Inability to autonomously cook and prepare meals | 1 if present, 0 if absent |
| Inability to autonomously do house chores | 1 if present, 0 if absent |
| Inability to autonomously do the laundry | 1 if present, 0 if absent |
| Inability to autonomously manage finances | 1 if present, 0 if absent |
| Inability to autonomously use the telephone | 1 if present, 0 if absent |
| Inability to autonomously use means of transportation | 1 if present, 0 if absent |
| Minimental State Examination score | 1 if < 18, 0.5 if 18 ≤ score < 23, 0.25 if ≤ 24 score < 27, 0 if ≥ 27 |
| Inability to autonomously bath | 1 if present, 0 if absent |
| Inability to autonomously dress | 1 if present, 0 if absent |
| Inability to autonomously use the toilet | 1 if present, 0 if absent |
| Incontinence | 1 if present, 0 if absent |
| Inability to autonomously move in and out from bed or chair | 1 if present, 0 if absent |
| Inability to autonomously feed him-/herself | 1 if present, 0 if absent |
| Usage of stick during walking | 1 if present, 0 if absent |
| Reporting dyspnoea | 1 if present, 0 if absent |
| Evidence of swollen legs at physical examination | 1 if present, 0 if absent |
| Reporting anxiety symptoms | 1 if present, 0 if absent |
| Self-reporting appetite loss | 1 if present, 0 if absent |
| Self-reporting sense of worthlessness | 1 if present, 0 if absent |
| Reporting pain | 1 if present, 0 if absent |
| Feeling older than actual age | 1 if present, 0 if absent |
| Self-reporting low quality of life | 1 if present, 0 if absent |
