# Supplementary material for: Comparing the prognostic value of geriatric health indicators: a population-based study
Source: BMC Med. 2019 Oct 2;17:185. doi: 10.1186/s12916-019-1418-2 (PMC6774220; doi:10.1186/s12916-019-1418-2)
Supplement: Supplementary file 2 — Table S2. Variables associated with indicator variable of missing values used during multiple imputation process. (DOCX 13 kb) [file 12916_2019_1418_MOESM2_ESM.docx]

**Table S2**: variables associated with indicator variable of missing values used during multiple imputation process

| Variable | Odds Ratio (95% Confidence Interval) |
| --- | --- |
| Age | 1.07 (1.05-1.08) |
| Frequent feeling of fatigue | 1.06 (0.76-1.48) |
| Being physically inactive | 2.85 (2.29-3.55) |
| Gender (Male = reference) | 1.46 (1.15-1.86) |
| Anemia | 1.23 (0.90-1.68) |
| Atrial fibrillation | 0.84 (0.58-1.21) |
| Blindness | 2.83 (1.65-4.83) |
| Bradycardias | 1.16 (0.57-2.34) |
| Heart valve diseases | 0.56 (0.31-1.03) |
| Cataract | 1.15 (0.76-1.73) |
| Cerebrovascular diseases | 1.52 (1.04-2.22) |
| Chronic kidney disease | 0.76 (0.58-0.98) |
| COPD and chronic bronchitis | 1.36 (0.87-2.10) |
| Deafness | 1.09 (0.78-1.52) |
| Dementia | 8.50 (4.99-14.50) |
| Diabetes | 0.94 (0.65-1.35) |
| Dyslypidemia | 0.65 (0.52-0.82) |
| Dorsopathies | 0.83 (0.55-1.26) |
| Ear, nose, and throat diseases | 0.55 (0.15-1.95) |
| Heart failure | 1.25 (0.86-1.81) |
| Inflammatory arthropaties | 0.89 (0.54-1.48) |
| Ischemic heart disease | 0.99 (0.73-1.34) |
| Parkinson and parkinsonism | 1.90 (0.81-4.43) |
| Peripheral neuropathy | 0.87 (0.61-1.24) |
| Solid neoplasm | 0.87 (0.61-1.24) |
| Count of chronic conditions | 1.10 (1.01-1.19) |
| Date of death | 0.84 (0.79-0.89) |
| 10-year mortality | 0.79 (0.52-1.22) |
