# Supplementary material for: Comparing the prognostic value of geriatric health indicators: a population-based study
Source: BMC Med. 2019 Oct 2;17:185. doi: 10.1186/s12916-019-1418-2 (PMC6774220; doi:10.1186/s12916-019-1418-2)
Supplement: Supplementary file 3 — Table S3. Areas under ROC curves for different indicators – complete dataset analyses. (DOCX 13 kb) [file 12916_2019_1418_MOESM3_ESM.docx]

|  | AUC (95%CI) |
| --- | --- |
| 3-year mortality | |
| Frailty index | 0.80 (0.77-0.83) |
| Frailty phenotype | 0.68 (0.63-0.73) |
| Health assessment tool | 0.86 (0.83-0.88) |
| Multimorbidity | 0.71 (0.68-0.73) |
| Walking speed | 0.84 (0.82-0.87) |
| 5-year mortality | |
| Frailty index | 0.80 (0.77-0.82) |
| Frailty phenotype | 0.66 (0.62-0.69) |
| Health assessment tool | 0.85 (0.83-0.86) |
| Multimorbidity | 0.72 (0.70-0.74) |
| Walking speed | 0.83 (0.81-0.85) |
| 1-year unplanned hospitalization | |
| Frailty index | 0.74 (0.71-0.77) |
| Frailty phenotype | 0.60 (0.56-0.64) |
| Health assessment tool | 0.73 (0.71-0.76) |
| Multimorbidity | 0.70 (0.67-0.72) |
| Walking speed | 0.72 (0.69-0.74) |
| 3-year unplanned hospitalization | |
| Frailty index | 0.73 (0.71-0.75) |
| Frailty phenotype | 0.60 (0.58-0.62) |
| Health assessment tool | 0.71 (0.69-0.73) |
| Multimorbidity | 0.68 (0.67-0.70) |
| Walking speed | 0.70 (0.68-0.72) |
| 2+ provider contacts | |
| Frailty index | 0.66 (0.64-0.68) |
| Frailty phenotype | 0.57 (0.55-0.59) |
| Health assessment tool | 0.63 (0.61-0.65) |
| Multimorbidity | 0.67 (0.65-0.68) |
| Walking speed | 0.62 (0.60-0.64) |

**Table S3:** Areas under ROC curves for different indicators (complete dataset)
