# Supplementary material for: Comparing the prognostic value of geriatric health indicators: a population-based study
Source: BMC Med. 2019 Oct 2;17:185. doi: 10.1186/s12916-019-1418-2 (PMC6774220; doi:10.1186/s12916-019-1418-2)
Supplement: Supplementary file 4 — Table S4. Areas under ROC curves for different indicators – imputed dataset analyses. (DOCX 13 kb) [file 12916_2019_1418_MOESM4_ESM.docx]

**Table S4**: Areas under ROC curves for different indicators

|  | AUC (95%CI) | p |
| --- | --- | --- |
| 3-year mortality | | |
| Frailty index | 0.84 (0.82-0.86) | < 0.001 |
| Frailty phenotype | 0.80 (0.78-0.82) | < 0.001 |
| Health assessment tool | 0.87 (0.85-0.88) | Ref |
| Multimorbidity | 0.71 (0.68-0.73) | < 0.001 |
| Walking speed | 0.85 (0.83-0.87) | 0.021 |
| 5-year mortality | | |
| Frailty index | 0.84 (0.82-0.86) | < 0.001 |
| Frailty phenotype | 0.79 (0.77-0.80) | < 0.001 |
| Health assessment tool | 0.86 (0.85-0.88) | Ref |
| Multimorbidity | 0.72 (0.70-0.74) | < 0.001 |
| Walking speed | 0.85 (0.83-0.86) | 0.006 |
| 1-year unplanned hospitalization | | |
| Frailty index | 0.73 (0.71-0.76) | 0.466 |
| Frailty phenotype | 0.68 (0.66-0.71) | < 0.001 |
| Health assessment tool | 0.73 (0.71-0.75) | Ref |
| Multimorbidity | 0.70 (0.67-0.72) | 0.009 |
| Walking speed | 0.72 (0.70-0.74) | 0.048 |
| 3-year unplanned hospitalization | | |
| Frailty index | 0.72 (0.70-0.73) | Ref |
| Frailty phenotype | 0.66 (0.64-0.68) | < 0.001 |
| Health assessment tool | 0.71 (0.69-0.73) | 0.790 |
| Multimorbidity | 0.68 (0.67-0.70) | 0.004 |
| Walking speed | 0.69 (0.68-0.71) | 0.098 |
| 2+ provider contacts | | |
| Frailty index | 0.64 (0.62-0.66) | 0.011 |
| Frailty phenotype | 0.58 (0.56-0.59) | < 0.001 |
| Health assessment tool | 0.62 (0.60-0.64) | < 0.001 |
| Multimorbidity | 0.67 (0.65-0.68) | Ref |
| Walking speed | 0.60 (0.59-0.62) | < 0.001 |
