# Supplementary figures and images for: Comparing the prognostic value of geriatric health indicators: a population-based study
Source: BMC Med. 2019 Oct 2;17:185. doi: 10.1186/s12916-019-1418-2 (PMC6774220; doi:10.1186/s12916-019-1418-2)

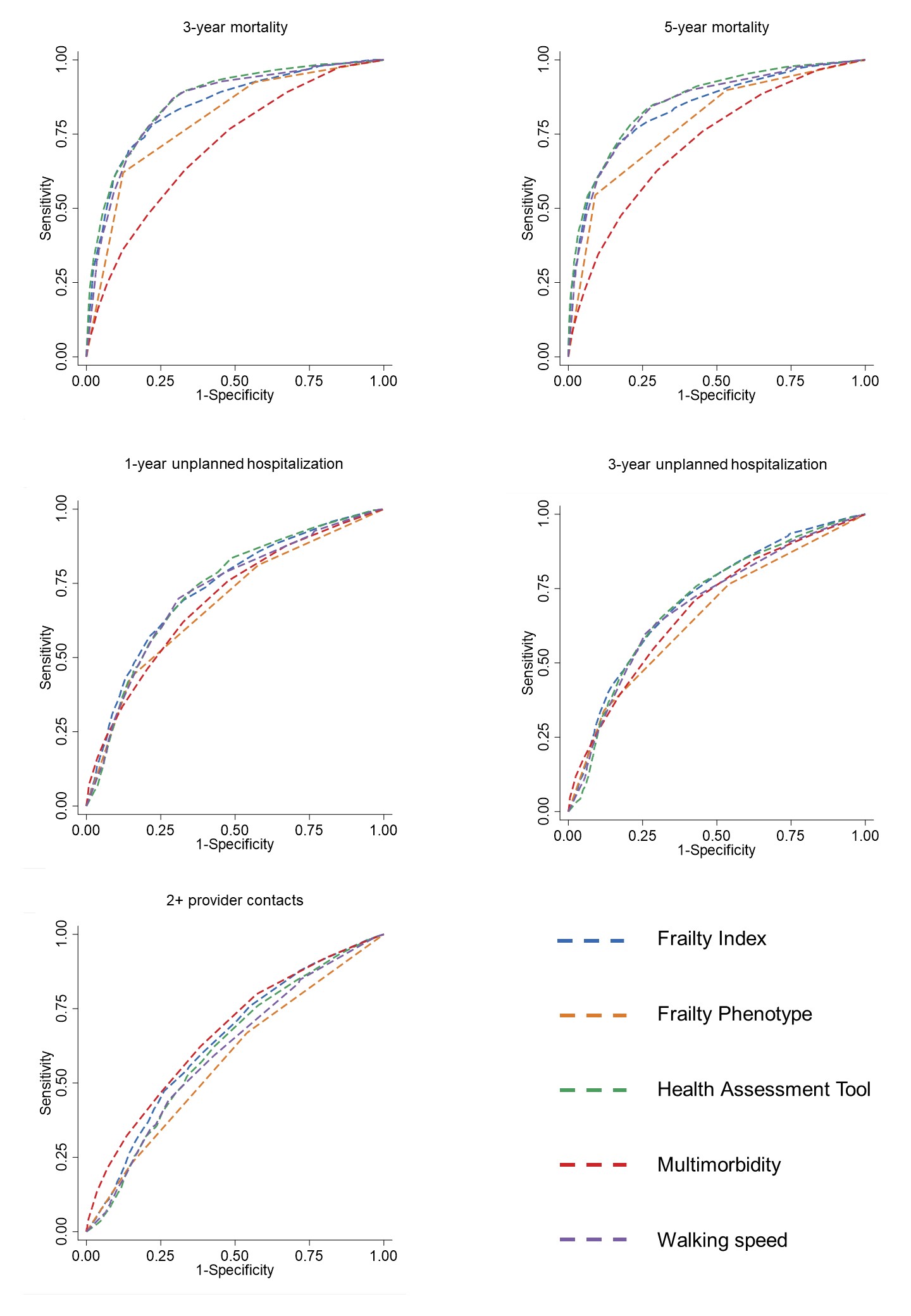

Supplement: Supplementary file 5 — Figure S1. ROC curves comparison. (JPG 241 kb) [file 12916_2019_1418_MOESM5_ESM.jpg]
