# Supplementary material for: Comparing the prognostic value of geriatric health indicators: a population-based study
Source: BMC Med. 2019 Oct 2;17:185. doi: 10.1186/s12916-019-1418-2 (PMC6774220; doi:10.1186/s12916-019-1418-2)
Supplement: Supplementary file 6 — Table S5. Areas under ROC curves for different indicators – analyses stratified by age. (DOCX 15 kb) [file 12916_2019_1418_MOESM6_ESM.docx]

**Table S5**: Areas under ROC curves for different indicator. Analyses stratified by age.

|  | AUC (95%CI)  < 78 years old | AUC (95%CI)  ≥ 78 years old |
| --- | --- | --- |
|  | **3-year mortality** | |
| Frailty index | 0.73 (0.66-0.80) | 0.78 (0.75-0.80) |
| Frailty phenotype | 0.69 (0.62-0.76) | 0.73 (0.70-0.75) |
| Health assessment tool | 0.74 (0.66-0.82) | 0.80 (0.78-0.82) |
| Multimorbidity | 0.65 (0.58-0.72) | 0.60 (0.57-0.63) |
| Walking speed | 0.73 (0.66-0.81) | 0.78 (0.75-0.80) |
|  | **5-year mortality** | |
| Frailty index | 0.73 (0.68-0.77) | 0.78 (0.76-0.81) |
| Frailty phenotype | 0.68 (0.63-0.73) | 0.73 (0.71-0.76) |
| Health assessment tool | 0.74 (0.69-0.79) | 0.80 (0.78-0.83) |
| Multimorbidity | 0.66 (0.61-0.71) | 0.62 (0.59-0.65) |
| Walking speed | 0.74 (0.69-0.79) | 0.78 (0.76-0.81) |
|  | **1-year unplanned hospitalization** | |
| Frailty index | 0.66 (0.62-0.71) | 0.68 (0.65-0.71) |
| Frailty phenotype | 0.60 (0.56-0.65) | 0.63 (0.60-0.66) |
| Health assessment tool | 0.68 (0.63-0.72) | 0.66 (0.63-0.69) |
| Multimorbidity | 0.65 (0.60-0.69) | 0.64 (0.60-0.67) |
| Walking speed | 0.65 (0.60-0.70) | 0.66 (0.63-0.69) |
|  | **3-year unplanned hospitalization** | |
| Frailty index | 0.66 (0.63-0.69) | 0.62 (0.60-0.65) |
| Frailty phenotype | 0.58 (0.55-0.61) | 0.60 (0.58-0.63) |
| Health assessment tool | 0.64 (0.60-0.67) | 0.60 (0.57-0.63) |
| Multimorbidity | 0.64 (0.60-0.67) | 0.60 (0.58-0.63) |
| Walking speed | 0.61 (0.58-0.65) | 0.60 (0.58-0.63) |
|  | **2+ provider contacts** | |
| Frailty index | 0.65 (0.63-0.68) | 0.55 (0.52-0.58) |
| Frailty phenotype | 0.56 (0.53-0.58) | 0.52 (0.50-0.55) |
| Health assessment tool | 0.61 (0.58-0.64) | 0.49 (0.46-0.52) |
| Multimorbidity | 0.66 (0.64-0.69) | 0.62 (0.59-0.65) |
| Walking speed | 0.59 (0.56-0.61) | 0.52 (0.49-0.55) |
